# Pharmacological Advances for Treatment in Hypertension

**DOI:** 10.3390/ph17010039

**Published:** 2023-12-27

**Authors:** Arquimedes Gasparotto Junior

**Affiliations:** Laboratory of Cardiovascular Pharmacology (LaFaC), Faculty of Health Sciences, Federal University of Grande Dourados, Dourados 79804-970, MS, Brazil; arquimedesgasparotto@gmail.com; Tel.: +55-67981159006

## 1. Introduction

Hypertension plays a significant role in the development of cardiovascular disease and renal diseases, which can heighten the likelihood of experiencing related conditions like myocardial infarction, stroke, and heart failure [1]. Currently, the treatment of hypertension basically involves renin-angiotensin system inhibitors (ACE inhibitors or AT1 receptor antagonists), inhibitors of the sympathetic autonomic nervous system (beta-adrenoceptor antagonists (‘beta blockers’), alpha 2 adrenoceptor agonists or alpha 1 adrenoceptor antagonists), calcium channel blockers, or diuretics [2]. Several studies have demonstrated that monotherapy to treat hypertension may be ineffective and could also lead to additional negative impacts on blood pressure management [3]. Furthermore, the exploration of novel drug targets in antihypertensive treatment is challenging. Until now, several new prototypes, such as aldosterone receptor antagonists, aminopeptidase A inhibitors, and vaccines, have been studied in phase I/II clinical trials. Furthermore, numerous new molecules have also been evaluated in animal studies [4]. These findings, along with a comprehension of their mechanisms of action, offer new perspectives for the treatment of hypertension. Nevertheless, it remains uncertain whether drugs that target newly identified targets are both efficient and selective. Therefore, it is important to research and discover new drugs, as well as new therapeutic targets, for the treatment of hypertension.

This Special Issue aims to compile and share the latest advancements in hypertension treatment research, focusing particularly on bioactive compounds that have health-promoting and therapeutic properties. Therefore, this information could be valuable in filling the knowledge gaps we currently have regarding the therapeutic abilities of novel synthetic or natural compounds. Six original scientific papers and two reviews examining the different aspects of hypertension treatment are included. These cover clinical management, innovative therapeutic options, and new animal models for preclinical research.

## 2. An Overview of the Published Articles

In the first study, Escudero Gómez (contribution 1) aims to determine the effect of COVID-19 on the metabolic profile of patients with hypertension. Specifically, the study looks at the correlation between COVID-19 and the plasmatic levels of losartan and its active metabolite (EXP3174), as well as other biochemical markers and blood pressure control. The results show that metabolomics is an important tool for evaluating the effectiveness of losartan pharmacotherapy and assessing the damage caused by COVID-19 and hypertension in hypertensive patients.

The article written by Moreno (contribution 2) showcases advancements in the development of an animal model that incorporates various cardiovascular risk factors, such as hypertension, hypothyroidism, and a high-fat diet. The experimental model induces dyslipidemia, impaired renal function, and hepatic steatosis, along with elevated levels of various inflammatory markers and increased serum oxidative stress. The findings support a feasible alternative model that includes multiple cardiovascular risk factors and resembles the conditions observed in hypertensive humans.

In the third study, Ho (contribution 3) presents the results of the synergistic effect of exercise combined with DIKTNKPVIF (DF) peptides in improving hypertension in spontaneously hypertensive rats. Exercise increases the expression of the cell-survival pathway proteins IFG1, PI3K, and AKT in a synergistic manner when combined with DF administration. Additionally, the administration of DF and exercise together leads to an increased expression of the pathway proteins AMPK, SIRT1, PGC-1α, and FOXO3. The data suggest that exercise, in conjunction with DF peptides, collaborates to reduce hypertension by activating the pathway of mitochondrial biogenesis.

The study by Qian (contribution 4) investigates whether adrenomedullin (ADM), an essential naturally occurring peptide, provides a protective role in the remodeling and functioning of the heart in rats with obesity-related hypertension (OH). The authors demonstrate that ADM reduces cardiac inflammation and oxidative stress by activating the receptor-Akt pathway, which, in turn, enhances cardiac remodeling and function in OH rats.

Furthermore, this special edition includes two reviews. The study performed by Remiszewski and Malinowska (contribution 5) investigates previous data that show the vasodilator response of (endo)cannabinoids. The researchers also propose that these compounds could be utilized in different forms of hypertension. Additionally, the review outlines all the publications discovered in the PubMed database concerning the extended use of (endo)cannabinoids in experimental models of systemic and pulmonary hypertension. The second review article, conducted by Cazarim (contribution 6), aims to conduct a systematic review with meta-analysis to investigate if medication therapy management by pharmaceutical care could improve the effectiveness of long-term clinical antihypertensive treatments.

## 3. Conclusions

This compilation of articles explores different approaches to the management of hypertension. This is also reflected in the different methodologies adopted for the studies, ranging from qualitative approaches to quantitative studies to assess the effectiveness of new therapies. The central framework of this special edition is formed by preclinical studies consisting of four articles, as well as clinical data involving hypertensive patients. This variety of topics highlights the relevance and importance of this special edition, presenting the reader with research focused on different contexts and providing a more comprehensive view of the research field. We encourage researchers to draw inspiration from this Special Issue in order to develop and validate new methods for the prevention and treatment of hypertension. This can serve as a complement or alternative to available treatments.

## References

[1] Rahimi K., Emdin C.A., MacMahon S. (2015). The epidemiology of blood pressure and its worldwide management. Circ. Res..

[2] Al Ghorani H., Götzinger F., Böhm M., Mahfoud F. (2022). Arterial hypertension—Clinical trials update 2021. Nutr. Metab. Cardiovasc. Dis..

[3] Carey R.M., Moran A.E., Whelton P.K. (2022). Treatment of Hypertension: A Review. J. Am. Med. Assoc..

[4] Gao Q., Xu L., Cai J. (2021). New drug targets for hypertension: A literature review. Biochim. Biophys. Acta Mol. Basis Dis..

